# Nature and Art

**Published:** 1873

**Authors:** Robert Battey

**Affiliations:** Rome, Georgia, Professor of Obstetrics in the Atlanta Medical College


### NATURE AND ART.

BY ROBERT BATTEY, M.D., ROME, GEORGIA,

*Professor of Obstetrics in the Atlanta Medical College.*

In the practice of the obstetrical art, as in most departments of operative medicine, it is exceedingly difficult to lay down any absolute rules for interference with the processes of nature. When all has been said, which it is proper to say, for the instruction and guidance of the inexperienced, discretion and good sound judgment must, after all, guide the practitioner, that he may *do the right thing, in the right way, and at the right time*, and more especially that he may sedulously guard himself against any officious interference which would be harmful in its results.

Trite and true as is the time-honored maxim, "a meddlesome midwifery is bad," it can by no means be said that the office of the *accoucheur* is a mere sinecure. There is undoubtedly much that he may do, and ought to do, in his ministrations, which is of service in any case, and of indispensable, yes *vital* service under certain conditions.

In the oldest work upon midwifery, printed in our language, we have the injunction of Thomas Rainalde: "The mydwyfe muste enstruct and comfort the partie, not onlye refreshyng her with goode meete and drinke, but alsoe with sweete wordes, gevyng her goode hope of a speedy deliveraunce, encouraging and enstomakyng her to patience and toleraunce, byddyng her to holde in breathe so much as she may," etc.

This counsel of old Thomas Rainalde is as good to-day as it was when first uttered, and gives the physician opportunity for usefulness when no active interference is required.

It is not our purpose to attempt to lay down any rules for the conduct of labor, but rather to offer our experience upon certain points, that it may assist others in the exercise of that wise discretion which grows out of much experience and multitude of counsel.

Under certain circumstances, when the *os* is well dilated or dilatable, and the labor appears to be impeded by toughness of the membranes, we are instructed by authority to rupture the membranes by art. This we have many times done in former years, and our experience has been that we have rarely, *very rarely*, seen the labor to be expedited in consequence; while, upon the other hand, we have in *very many* instances had cause to regret the interference. On several occasions we have delivered the ovum entire, and torn open the membranous sac to remove a healthy and vigorous child, and in such cases the labor has not been tedious nor unduly painful. Still oftener have we had the membranes torn loose from the placenta, and delivered enveloping the fœtal head.

It is unquestionably the spirit of *accoucheurs* of the present day to resort to delivery by *forceps* much more frequently than was formerly deemed justifiable. By this instrument, in skillful hands, very many children are rescued which would otherwise inevitably perish; very many mothers are saved long hours of untold suffering, to say nothing of the baneful sequences of exhaustion and long and severe pressure upon the pelvic tissues, sloughing, rectal and vesical fistulæ, etc.

Undoubtedly the operator ought to be well and truly instructed in the use of the instrument upon the phantom before essaying its use in the living body; quite as certain is it that no practitioner ought to esteem himself efficient in the art of obstetrics who does not possess this necessary skill.

Some years since a lady, who had been seven times delivered of still-born children, removed her residence that she might command better skill and better *luck* in her eighth confinement. She brought a letter from her former physician expressing the opinion that she could not bear a living child at term, and recommending premature delivery. At the accouchment, at full term, the child presenting by the breach, all went well; nothing was required but to turn the head out of the vagina, and a fine large child was safely delivered. In her ninth labor, the child presented by the vertex, and she delivered herself promptly before her physician could reach her. She now has four or five hearty, robust children.

Another lady, who had borne three still-born children, was advised by her physician that she was "too close made to bear a living child." Her fourth labor was natural every way, and required no assistance whatever.

These cases excite a suspicion of *meddlesome midwifery*.

We have found it always advisable to determine the presentation and position as early in the labor as is practicable. When this has been achieved, all subsequent "touching" is an evil which is to be avoided, and only resorted to when it becomes a *necessary* evil.

